# Companionship in Hospital Medicine: Expanding Care Through Community Volunteer Visitation

**DOI:** 10.7759/cureus.103979

**Published:** 2026-02-20

**Authors:** Zaki Khera, Bilal Irfan

**Affiliations:** 1 Department of Psychiatry, University of Michigan, Ann Arbor, USA; 2 Center for Surgery and Public Health, Brigham and Women's Hospital, Boston, USA; 3 Center for Bioethics, Harvard Medical School, Boston, USA

**Keywords:** community program, hospital medicine, patient visitation, religious accomodation, volunteer visits

## Abstract

Hospital medicine increasingly acknowledges the shaping of inpatient outcomes by not only biomedical interventions, but also emotional and spiritual well-being. This piece explores how community partnerships can complement existing spiritual care services when chaplaincy capacity is limited, utilizing the example of the hospitalized Muslim patient population. Muslim patients often face persistent gaps in meeting their religious and practical needs during hospitalization, including limited access to Muslim chaplains, prayer spaces, and their basic dietary needs. Building on these observations and the general literature on the negative impact of isolation in hospital settings, this piece argues for considering volunteer spiritual companionship as a pragmatic equity strategy presented in the form of a structured Muslim volunteer visitation framework developed through the collaboration of hospitals and communities. This approach offers a transferable template for extending culturally responsive companionship to other patient communities. Evaluation criteria for the implementation and success of such a program can include patient-reported loneliness, perceived spiritual support, overall satisfaction with their inpatient care, and improvement tracked over time using relevant spiritual assessments.

## Editorial

Hospital medicine has long affirmed that the welfare of the patient is the supreme law. Yet contemporary inpatient care is frequently organized around efficiency, diagnostics, and therapeutics, constraining clinical attention to only physiologic targets. In practice, this emphasis may leave emotional and spiritual concerns under-addressed, particularly for patients whose sources of meaning and coping are closely tied to community, ritual practice, and faith-based support. High-quality care is often viewed as prioritizing safety, efficacy, timeliness, efficiency, equity concerns, and patient-centered approaches, yet this can risk neglecting to actualize patients’ cultural and spiritual needs, especially among certain minority communities. The hospitalized Muslim patient population can be utilized as a case example of precisely where spiritual needs are often neglected, offering an opportunity to illustrate how religiously concordant companionship can be operationalized through a scalable, community-partnered approach. 

Several challenges surface in providing patient-centered care to Muslim patients. Muslim patients are heterogeneous in language, cultural practice, and religious observance, and their hospital experience may be shaped by issues relating to linguistic barriers, differing expectations of modesty, family involvement, dietary restrictions, fasting decisions, access to prayer spaces and devotional resources, and limited access to religiously concordant spiritual care. These spiritual and social needs intersect with communication and trust, particularly when patients view their values as misunderstood or disregarded. Traditionally, chaplaincy services have often served as an institutional mechanism to address these very needs, supporting patients in their ability to practice their faith while quelling spiritual distress, isolation, or conflict through conversation. One study found that patients who had seen chaplains trained in compassion-centered spiritual health had lower post-consult depression scores than patients who were seen by chaplains without such training [1]. Chaplains can work as liaisons who help care teams facilitate culturally sensitive conversations when distress or grief complicate clinical care, particularly in end-of-life cases. National data describing the healthcare chaplaincy workforce in the United States suggest that hospital spiritual care staffing remains predominantly Christian, with very limited Muslim representation among board-certified chaplains [2]. This limitation does not imply that non-Muslim chaplains are unable to provide high-quality spiritual care, but it does suggest that Muslim patients may have fewer opportunities to access faith-congruent counsel and culturally fluent communication at the bedside [2].

The clinical relevance of loneliness is increasingly difficult to dismiss. Social isolation in inpatient settings has been associated with higher levels of psychological distress and with downstream risks that include lapses in supportive care [3]. A systematic review of isolation practices in hospitalized patients reported consistent adverse psychosocial effects, including higher rates of depression, anxiety, anger/hostility, and reduced self-esteem, while also identifying safety concerns linked to reduced contact and monitoring [3]. Of course, isolation is not equivalent to loneliness, but both phenomena underscore the importance of presence, listening, and relational continuity as clinically meaningful. For Muslim patients, unmet spiritual and practical needs may compound these risks. Emerging evidence also indicates substantial gaps in the hospital’s capacity to meet Muslim patients’ religious and spiritual needs [4]. In a national survey of American Muslims, nearly all respondents rated fulfillment of religious needs as important during hospitalization, yet reported limited access to practical accommodations such as neutral prayer spaces and halal food [4]. When these gaps coincide with the limited availability of Muslim chaplaincy, patients may be left without reliable channels for spiritual reassurance and reduced confidence that the hospital environment recognizes their values, instead feeling misunderstood or even marginalized. As a result, the therapeutic relationship may suffer, trust may erode, and communication may narrow. Clinicians, despite being well-intentioned, could be perceived as discriminatory simply from their lack of understanding of basic Islamic teachings, including things like fasting, prayer, halal food, and hijab [2,3].

Given workforce, language, and cultural constraints, it is unlikely that chaplaincy pipelines alone will rapidly meet the demand for culturally concordant spiritual support across diverse patient populations. Volunteer-driven visitation programs offer a complementary approach that can expand relational and spiritual presence without substituting for clinical care or licensed chaplaincy. Of course, it is important to specify that volunteers cannot function as clinicians, provide medical advice, or even replace the role of chaplaincy consultation, especially in the presence of complex ethical conflicts. Instead, they may serve as trained companions who offer attentive listening, emotional presence, and patient-directed support for devotional practice if requested.

For such programs to be acceptable for hospitals, ethical safeguards and careful regulation are crucial. When properly structured, volunteer programs can be designed to prioritize patient consent, confidentiality, infection control, and defined boundaries around physical touch, documentation, and interaction with clinicians. Clear escalation pathways are essential. Volunteers should be trained to alert nurses, physicians, or chaplains when patients demonstrate safety concerns, severe distress, or make requests that exceed their scope. Due to the diversity of religious law and interpretation within the Muslim community, they should also be trained to practice humility and restraint when navigating controversial or theologically complex conversations (e.g., providing religious rulings). Overall, volunteer implementation should prioritize protecting patients from misinformed guidance and institutions from role confusion.

The long-term care literature describes structured models for recruiting, training, and sustaining volunteer spiritual care visitors, emphasizing reflective listening, grief support competencies, and clear supervision frameworks [5]. These features translate well to hospital environments where brief but intentional companionship may reduce perceived isolation and strengthen patients’ sense of dignity. The Anees Model is one example of a Muslim-oriented volunteer visitation framework designed to standardize community-based spiritual companionship in partnership with hospital spiritual care departments. The Anees program addresses demand for chaplaincy-adjacent support among Muslim patients by working closely with Muslim communities and spiritual care stakeholders across the United States. Volunteers may be recruited through local mosques, universities, medical schools, and resident physician networks, which can diversify language capacity and improve cultural responsiveness [6].

Operationally, candidates complete hospital onboarding requirements, such as background checks, immunizations, and other institutional clearances, alongside specialized training. Training is delivered through asynchronous modules developed with chaplains, clinicians, and imams familiar with Muslim patient care. Core content emphasizes active and reflective listening, grief literacy, modesty considerations, patient confidentiality, and appropriate use of devotional resources when requested by patients (such as supplication cards, prayer rugs, or religious texts). Shadowing opportunities with board-certified chaplains and experienced volunteers can further strengthen bedside readiness and reinforce boundaries, communication standards, and escalation to clinical teams when concerns emerge [6].

Because it leverages existing volunteer infrastructures, a program of this type may be implemented without extensive structural redesign. However, implementation challenges with such a program should also be acknowledged. Liability concerns, variability in community engagement, volunteer burnout, and a lack of institutional support can constrain sustainability or even prevent a program from starting in the first place. These risks can be mitigated through formal memoranda of understanding with hospitals, partnerships with national chaplaincy organizations, periodic retraining and follow-up with volunteers, supervision by spiritual care leadership, and defined criteria for initial screening. 

The underlying logic of structured companionship extends beyond just Muslim patient care. Faith-congruent companionship can be built for other communities using similar safeguards, training expectations, and professional oversight, thereby advancing equity through scalable relational care. If hospital medicine aspires to healing that is not only physiologic but also psychological and spiritual, companionship should be treated as a legitimate domain for quality improvement and research. Structured visitation programs can formalize presence as a therapeutic adjunct, while honoring institutional standards and patient autonomy. For the patient, compassionate companionship may restore meaning and reduce perceived abandonment within an unfamiliar clinical environment. For the care team, it offers a practical mechanism to extend culturally responsive support at the bedside.
